# Commencement Exercises

**Published:** 1903-04

**Authors:** 


### COMMENCEMENT EXERCISES.

### ONTARIO VETERINARY COLLEGE.

The closing exercises of this College for the season 1902-3 were held March 26th, with the Principal, Dr. Andrew Smith, presiding, supported on the platform by his Worship the Mayor; Dr. Rutherford, Chief Veterinary Inspector, Ottawa; Col. Lloyd, Newmarket; Dr. Cowan, Guelph; Dr. Brenton, Detroit, Mich.; Dr. J. F. Duncan; Professors Lang and Amyot, Toronto University, and Dr. D. King Smith.

After a few introductory remarks by the Principal, the Mayor addressed the graduating class, numbering over one hundred, gathered from all parts of the world. His Worship congratulated Dr. Smith upon the high position attained by the College. He warned the young men that in leaving college they were but entering upon life and that experience in the profession they were entering upon—one of the noblest known—would teach them many things not to be found in books.

Dr. Duncan presented the prizes, which were very numerous and valuable. In doing so he remarked upon the excellence of the class.

After the presentation, Dr. Smith said he was very pleased to have present Dr. Rutherford, Chief Veterinary Inspector of the Dominion, who graduated from the College.

Professor Lang congratulated the Principal on the success of the College. He referred to the struggle every young practitioner experienced, but said that everything comes to him who knows how to wait. Some men were students all their lives, and in no profession was there more to be found out. Dr. Amyot cautioned the student not to stop working, and, above all, to continue his study of physiology, so that he might prescribe rationally. Col. Lloyd and Dr. Cowan, two of the examiners, testified that the class of 1903 was the best that had come under their notice. The latter referred to the fact that nearly every State in the Union and every civilized country in the world, as well as every city and province in Canada, were represented in the classes of the College.

Dr. Brenton, of Detroit, said that, as proof of his confidence and appreciation of the College, from which he graduated twenty-three years ago, his son was one of this year's graduating class.

Dr. Rutherford, Chief Veterinary Inspector for the Dominion, who graduated twenty-four years ago, delivered at once a practical, instructive, and amusing address. He had not attended the closing exercises for twenty years. He was gratified to notice the good feeling that evidently existed between the teaching staff and the students. He congratulated Dr. Smith upon the hardy annuals his examiners were, the same that acted now having heckled him in his student days. He repeated the advice of the Mayor as to observation, saying that in no profession was it more valuable, as animals could not talk like human beings. There was a new and useful field open to them—the field of controlling diseases.

Mr. F. B. Lambie, gold medallist, presented the Principal with a large picture of the graduating class of 1903, and, with enthusiastic cheers for the King, Dr. Smith, and the College, the proceedings closed.

Following are the results:

*Graduates.* A. E. Bowman, St. Thomas; William Boyd, London; G. W. Brashear, Newtown, Ky.; W. L. Brenton, Detroit, Mich.; A. W. Busselle, Guelph; A. H. Chamberlain, Lexington, N. Y.; D. H. Chase, Flint, Mich.; J. T. Chorlton, Providence, R. I.; G. A. Coates, Port Perry; John Coyle, Wilmington, Ohio; H. J. Culp, Orangeville; W. E. Coover, Muncie, Ind.; A. P.

Drew, Providence, R. I.; L. A. Evans, Sawyerville, P. Q.; William M. Evans, Elizabeth, N. J.; J. M. Fawcett, Drayton; Alberto C. Fernandez, Buenos Ayres, Argentine; F. J. Fischer, Petersburg, Va.; D. B. Fraser, Forest; C. J. Gillen, Ottawa, Ill.; W. E. Grace, Toledo, Ohio; J. A. Graham, Elkhart, Ind.; J. W. Haffer, Paterson, N. J.; F. Narburn, Mitchell; William J. Hartman, Ladner, B. C.; William Henderson, Toronto; H. E. Houze, Atwood; E. H. Ives, New Hudson, N. Y.; C. B. Kern, Shimerville, Pa.; F. B. Lambie, Brussels; W. J. Lee, Vasey; C. A. Leslie, Belvidere, Neb.; E. W. MacKay, Sawyer; William J. McLevey, Florence; Alexander MacMillan, Sonya; R. T. Mack, South Bend, Ind.; H. S. Maxwell, Salina, Kansas; John H. Meany, Athol, Mass.; F. C. Miller, Dubuque, Iowa; F. D. Monell, Waterbury, Conn.; Vincente Ocampo, Buenos Ayres, Argentine; William O. Oliver, Marinette, Wis.; Byron A. Owens, Massillon, Ohio; J. H. Part, Leigh, Lancashire, England; H. Pomfret, Burnley, Lancashire, England; M. Porterfield, Clifford; T. F. Quinn, Jr., North Adams, Mass.; A. A. Reinhardt, Apple Creek, Ohio; L. J. Richards, Granville, Ohio; George W. Rogers, Rochester, N. Y.; J. C. Rusk, Clifford; W. N. Russell, Buffalo, N. Y.; Fred. W. Schweinler, Jefferson, Wis.; R. A. Sibley, Waltham, Mass.; B. C. Smith, Brigden; William A. W. Sparling, Toronto; E. R. Struve, Parsons, Kansas; Thomas O. Sykes, Sykesville, Pa.; H. J. Taylor, Guelph; L. H. Thurston, Parsons, Kansas; G. R. Tomlinson, Orion, Ill.; C. Van Vlaanderen, Paterson, N. J.; W. M. Walker, Brooklyn, N. Y.; George C. Webb, Akron, Ohio; Guy N. Welch, Groton, Vt.; H. L. Williams, Granville, Ohio; T. W. Wilson, Atwood.

*Disease and Treatment.* First prize, silver medal: J. H. Part. Second prize: F. B. Lambie. Third prize: C. A. Leslie, C. B. Kern, equal.

Honors: A. H. Chamberlain, J. Chorlton, G. Coates, A. P. Drew, A. Fernandez, F. Fischer, D. B. Fraser, J. W. Haffer, W. A. Henderson, E. H. Ives, W. J. Lee, Alexander MacMillan, R. Mark, J. H. Meaney, F. C. Miller, V. Ocampo, H. Pomfret, T. Quinn, L. Richards, E. B. Struve, T. O. Sykes, H. J. Taylor, G. C. Webb, G. Welch, H. Williams.

*Materia Medica.* First prize: Ch. Kern, A. H. Chamberlain, equal. Second prize: H. Williams. Third prize: J. Chorlton.

Honors: W. Coover, D. Fraser, T. Fawcett, T. Hartman, H. Houze, E. Ives, C. Leslie, W. Lee, F. B. Lambie, Alexander MacMillan, J. H. Part, W. Sparling, T. O. Sykes, H. Taylor, W. Walker, F. Wilson, G. Webb.

*Chemistry.* First prize: F. B. Lambie, J. H. Part, equal. Second prize: D. Fraser, C. A. Leslie, equal.

Honors: G. Coates, J. Fawcett, A. Fernandez, W. Hartman, W. A. Henderson, E. Ives, W. J. Lee, J. Meaney, Alexander MacMillan, V. Ocampo, J. Rusk, R. A. Sibley, T. O. Sykes, J. Wilson, G. Welch.

*Pathology.* First prize: F. B. Lambie. Second prize: C. A. Leslie. Third prize: V. Ocampo.

Honors: George Coates, D. B. Fraser, A. Fernandez, W. Grace, W. A. Henderson, F. Harburn, E. Ives, Charles Kern, Wm. Lee, Alexander MacMillan, H. Pomfret, J. H. Part, J. Rusk, W. Sparling, R. Sibley, E. Struve, B. C. Smith, H. J. Taylor, C. Van Vlaanderen, H. Williams, G. Welch.

*Physiology.* First prize: F. B. Lambie. Second prize: J. H. Part. Third prize: C. A. Leslie.

Honors: Alberto Fernandez, D. Fraser, W. Hartman, E. Ives, C. B. Kern, Vincente Ocampo, H. Pomfret, G. Rogers, T. O. Sykes, H. J. Taylor, T. Wilson, W. Walker.

*Anatomy.* First prize, silver medal: C. A. Leslie. Second prize: F. B. Lambie. Third prize: C. B. Kern.

Honors: A. H. Chamberlain, G. A. Coates, W. E. Coover, A. P. Drew, D. B. Fraser, J. W. Haffer, W. A. Henderson, F. Harburn, W. J. Hartman, E. H. Ives, W. J. Lee, E. W. McKay, A. MacMillan, J. H. Meany, F. C. Miller, F. D. Monell, V. Ocampo, J. H. Part, H. Pomfret, M. Porterfield, T. Quinn, J. C. Rusk, W. A. W. Sparling, E. R. Struve, T. O. Sykes, H. J. Taylor, R. Tomlinson, C. Van Vlaanderen, W. M. Walker, George Webb, H. L. Williams, T. W. Wilson.

*Entozoa.* First prize: W. J. Lee and J. C. Rusk, equal.

Honors: W. L. Brenton, G. W. Busselle, D. H. Chase, G. A. Coates, F. J. Fischer, D. B. Frazer, Foster Harburn, J. W. Haffer, W. A. Henderson, W. J. Hartmann, E. H. Ives, C. B. Kern, C. A. Leslie, F. B. Lambie, J. H. Meany, V. Ocampo, H. Pomfret, G. W. Rogers, L. J. Richards, W. A. W. Sparling, R. A. Sibley, B. C. Smith, T. O. Sykes, H. J. Taylor, H. L. Williams, G. W. Welch.

Dissected specimens: Gold medal given by the Toronto Industrial Association; awarded to J. H. Part. Second prize: W. A. W. Sparling. Third prize: J. C. Rusk.

Best general examination: Gold medal, given by the Ontario Veterinary Association; awarded to F. B. Lambie.

Honors: C. B. Kern, C. A. Leslie, J. H. Part.

*Primary Examinations.* Anatomy: Charles W. Bandy, C. F. W. Bauer, Anson W. Beach, William J. Boddy, Thomas E. Bowers,

George Bulgin, James McDermid, J. Stelck Mack, J. H. Mann, P. E. Pallister, T. A. Parker, J. C. Taylor.

*Materia Medica.* P. Ernest Pallister, Daniel J. Seifert, John C. Taylor.

*Disease and Treatment.* First prize: B. Eddy. Second prize: G. Deming. Third prize: C. Mix.

Honors: J. Badgley, T. Bailey, C. Boissieu, G. Brewster, F. Burt, A. B. Campbell, A. R. Coleman, F. Consaul, G. Cunningham, J. Dilley, J. H. Faust, G. D. Fisher, J. Frank, C. Grantham, G. Johnston, T. Krey, A. A. Lockhart, W. McFadden, A. Mair, H. Mallory, Walter Martin, F. Mathews, A. Munn, S. Murray, T. Nichol, C. A. Philps, C. Randall, Olaf Reed, S. Robinson, T. Scrivener, W. Symms, A. Sexsmith, W. Stuart, A. Van Cleaf, W. Warnock, C. Weagley, F. Whele, W. Zirkle.

*Anatomy.* First prize, silver medal: Harvey Malloy. Second prize: S. A. Deming. Third prize: A. R. Coleman, Jr.

Honors: Charles Boissiere, J. A. Dilley, G. D. Fisher, J. W. Frank, G. A. Johnston, W. Kinie, A. A. Lockhart, A. M. Mair, Walter Martin, F. V. Mathews, C. C. Mix, A. A. Munn, S. Murray, T. Screvener, A. B. Sexsmith, W. P. Stuart, W. W. Warnock, C. B. Weagley, F. A. Wehle, W. W. Zirkle.

*Physiology.* First prize, silver medal: G. Deming. Second prize: A. R. Coleman. Third prize: C. Grantham.

Honors: C. L. Boissiere, T. Bailey, J. Frank, D. Fisher, W. Kime, T. Krey, A. A. Lockhart, A. Mair, Walter Martin, F. Mathews, S. Murray, T. Scrivener, W. Warnock.

*Chemistry.* First prize: A. R. Coleman. Second prize: C. L. Boissiere. Third prize: A. A. Lockhart.

Honors: G. Brewster, E. Beavers, T. Bailey, G. Cunningham, G. Deming, E. Daubert, G. Fisher, J. Frank, C. Grantham, G. Johnson, T. Krey, W. Kine, E. Lathrop, W. Martin, H. Malloy, A. Mair, S. Murray, C. Mix, J. Martin, G. Mathews, A. Munn, O. Reed, William Randall, A. Van Cleaf, W. Warnock, J. White, C. Weagley, W. Zirkle.

*Pathology.* First prize: A. R. Coleman. Second prize: C. L Boissiere. Third prize: W. Martin.

Honors: F. Burt, L. Burr, G. Brewster, T. Bailey, G. Cunningham, C. Dauber, J. Dilley, G. Deming, M. Faust, G. Fisher, J. Frank, C. Grantham, J. Jervis, William Kime, T. Krey, A. Lockhart, C. Lathrop, C. Mix, H. Malloy, J. Martin, A. Mair, S. Murray, F. Matthews, R. McKenzie, C. Philps, O. Reed, William Randall, William Symms, W. Stuart, T. Scrivener, A. Van Cleaf, F. Wehle, F. Woolley, W. Warnock, C. Weagley, W. Zirkle.

